# A Single‐Center, Open‐Label Study to Evaluate the Efficacy and Tolerability of Retinal Encapsulated in a Novel Biomimetic Exosome in the Treatment of Mild‐To‐Moderate Facial Photodamage

**DOI:** 10.1111/jocd.70755

**Published:** 2026-02-24

**Authors:** Michael H. Gold, Kaitlyn M. Enright, Glynis Ablon, Sheila Barbarino, Suneel Chilukuri, Doris Day, David J. Goldberg, Ted Lain, Ava Shamban, Todd Schlesinger

**Affiliations:** ^1^ Gold Skin Care Center and Tennessee Clinical Research Center Nashville Tennessee USA; ^2^ Klynical Consulting & Services Quebec Canada; ^3^ Ablon Skin Institute & Research Center California USA; ^4^ Barbarino Surgical Arts Austin Texas USA; ^5^ Refresh Dermatology Houston Texas USA; ^6^ Day Dermatology & Aesthetics New York New York City USA; ^7^ Cosmetic Dermatology & Clinical Research, Schweiger Dermatology Group New York New York City USA; ^8^ Sanova Dermatology Austin Texas USA; ^9^ AVA MD Medical & Cosmetic Dermatology California USA; ^10^ Clinical Research Center of the Carolinas Charleston South Carolina USA

**Keywords:** anti‐aging, photo aging, retinol, skincare, tretinoin, vitamin A

## Abstract

**Background:**

Numerous retinoid‐based skincare products are available over‐the‐counter for photodamaged and photoaged skin, but are associated with objective (e.g., erythema, dryness, flaking, edema) and subjective (e.g., itching, tingling, stinging, burning) tolerability issues. A novel retinal formulation was developed using an engineered biomimetic vegan exosome as a delivery system. The formulation also contains proprietary and potent hyaluronic acid, as well as plant‐based actives commonly used in traditional herbal medicines for their anti‐inflammatory and antioxidant properties.

**Aims:**

Evaluate the efficacy and tolerability of this biomimetic exosome‐encapsulated retinal product for treatment of mild‐to‐moderate facial photodamage.

**Patients/Methods:**

Twenty females aged 35 to 65 years, with Fitzpatrick skin types I‐VI, mild‐to‐moderate facial fine lines and wrinkles, and mild‐to‐moderate facial photodamage, were assessed at screening/baseline and weeks 2, 4, 8, and 12. Investigator‐ and participant‐assessed evaluations were performed at each visit. The primary objective was to demonstrate a decrease in the appearance of fine lines and wrinkles, erythema, as well as a reduction of overall facial photodamage after a 12‐week topical regimen. The secondary objective was to measure the frequency of adverse events throughout the study.

**Results:**

Statistically significant improvements in erythema, skin tone, skin texture, and lines/wrinkles were observed at all follow‐up visits, compared to baseline. At weeks 8 and 12, all participants (100%) displayed improvements in facial aesthetics. Most participants (19/20; 95.00%) were satisfied with the product and would recommend it to others. No product‐related adverse events were reported.

**Conclusions:**

Daily use of this “hydrating retinal” improved signs of facial photodamage with none to mild signs of skin irritation.

## 1 Introduction

Photoaging is the most significant contributing factor to facial aging and is among the most prevalent skin conditions [1, 2]. Skin features associated with photoaging include fine lines, coarse wrinkles, mottled/uneven pigmentation, loss of translucency and radiance, rough texture, decreased elasticity, dehydration, and erythema, among others [1, 2, 3, 4]. Retinoids are one of the most common topical treatments to prevent and treat facial photoaging [5, 6]. However, their use is often associated with retinization, a skin condition characterized by a collection of adverse effects, including erythema, peeling, dryness, and burning/stinging [4, 7]. Recently, a novel retinal formulation has been created using an innovative delivery system of an engineered biomimetic exosome (113 nm) (a functional signaling molecule) that also includes *Centella asiatica* , a plant used in traditional herbal medicines for its anti‐inflammatory and healing effects [8, 9, 10]. Additionally, the formulation contains a proprietary hyaluronic acid, manufactured with anion exchange, that contains both low (100KDa) and high molecular weights (2000KDa).

A variety of exogenous and endogenous nanomaterials have been developed as biological carriers (e.g., liposomes, micelles, peptides) [11, 12, 13]. Biomimetic exosomes, which are engineered nanoparticles that mimic the structure and function of natural exosomes, address several limitations of natural exosomes for therapeutic use [14]. Comparatively, biomimetic exosomes have excellent biocompatibility, low immunogenicity, low toxicity, and the ability to overcome biological barriers [14, 15, 16]. Encapsulating retinal in intentionally engineered biomimetic exosomes offers many benefits. For example, synthetic exosomes can be utilized as targeted delivery systems to activate a specific immune cascade [17]. Compared to traditional methods, exosomes can deliver the retinal to deeper skin layers, where they are more effective [18]. Encapsulation can also limit the severity of skin irritation typically associated with retinal, even at high concentrations. In this proprietary formulation, the incorporation of *Centella asiatica* into the exosome provides additional anti‐inflammatory and antioxidant benefits and helps retain moisture and strengthen the skin's natural barrier [8]. This formulation may provide for the most potent, yet tolerable, non‐prescription retinoid for the treatment of photo aged skin.

This study aimed to evaluate the efficacy and tolerability of a biomimetic exosome encapsulation delivery system for a retinal (retinaldehyde) product when used in the treatment of mild‐to‐moderate facial photodamage after 12 weeks of once daily topical treatment. Tolerability was assessed throughout the study.

## 2 Materials and Methods

### 2.1 Overall Study Design

Twenty participants were enrolled in this open‐label, single‐center study. After participants signed the informed consent form (ICF) and had been qualified for participation, they had their photographs taken before the first application of the investigational retinal product, which was first applied in the office by the study coordinator at visit 1, to ensure no adverse reactions (e.g., irritation, sensitization [contact allergy]). The product was then applied once daily in the evening after cleansing and before moisturizing. In the morning the routine was cleanser, moisturizer and sunscreen. The cleanser (Prelude Hydrinity Accelerated Skin Science, Monroe, LA, USA) and moisturizer (Cetaphil Daily Hydrating Lotion, Texas, USA) were distributed to the participants to ensure enough supply for twice daily use throughout the study. The patients were instructed to use their preferred sunscreen. The study coordinator educated participants on the proper application of the test product and reviewed the instructions for the take‐home diary. Participants kept their diaries for the duration of the study and returned them to the study coordinator at the end of study (EOS) visit. Throughout the study, participants were required to maintain their current brand of makeup and refrain from other cosmetic treatments or actives. Participants returned to the research center for follow‐up visits at weeks 2, 4, 8, and 12.

### 2.2 Description of Study Population

#### 2.2.1 Eligibility Criteria

Twenty (20) healthy females between the ages of 35 and 65 years, with Fitzpatrick skin type I‐VI, mild‐to‐moderate facial fine lines and wrinkles, and mild‐to‐moderate facial photodamage, were recruited to participate in this study. Although there was no eligibility restriction based on self‐identified race, this information was collected for statistical purposes as previous research had demonstrated race‐based differences in the tolerability of topical retinoids. Lists of eligibility criteria were used to determine which individuals were eligible for participation in the study. Inclusion criteria (Figure S1) defined the characteristics that made an individual suitable for the study, while exclusion criteria (Figure S2) specified the characteristics that disqualified them. At screening, the Investigator ensured that participants met all required eligibility criteria before enrolment and proceeding to the baseline visit.

### 2.3 Study Procedures

#### 2.3.1 Informed Consent/HIPAA/Photo Release

An Informed Consent Form (ICF) that included all the relevant elements required by the Food and Drug Administration (FDA) or state regulations was provided to each prospective study participant at screening and before enrollment into the study. As an addendum to the ICF, a photo release document specifically addressed the use of participant photographs. It outlined the specific purposes for which the images would be used (e.g., marketing, education, research). To ensure compliance with U.S. federal law and relevant regulations regarding the use of participant photos that contain protected health information (e.g., identifiable features), a Health Insurance Portability and Accountability Act (HIPAA) authorization accompanied the photo release. Following the informed consent process, the participant was asked to document consent by signing and dating in the appropriate areas of the ICF. The Investigator or designee also signed and dated the ICF.

#### 2.3.2 Fitzpatrick Wrinkle Assessment Scale

The Investigator used the Fitzpatrick Wrinkle Assessment Scale (FWAS) to evaluate the degree of elastosis (mild, moderate, severe). Mild elastosis is characterized by skin with fine texture changes and subtly accentuated skin lines. Moderate elastosis represents skin with distinct papular elastosis (individual papules with yellow translucency under direct lighting) and dyschromia. Severe elastosis represents skin with multipapular and confluent elastosis (thickened, yellow and pallid) approaching or consistent with cutis rhomboidalis. Only participants with mild‐to‐moderate facial skin elastosis qualified for the study.

#### 2.3.3 Glogau Wrinkle Grade

The Glogau Wrinkle Scale (GWS) measures the severity of wrinkles and photoaging, categorizing skin types into four distinct groups. Skin Type I presents without wrinkles, no‐to‐minimal discoloration or wrinkling, and no keratoses. Skin Type II presents with wrinkling in the skin with movement, slight lines near the mouth and eyes, and no keratoses. Skin Type III presents with wrinkles at rest, noticeable discolorations, and visible keratoses. Skin Type IV presents with wrinkles throughout (make‐up appears to cake and crack when applied), gray or yellow discoloration of the skin, and a history of prior skin cancer. In the present study, the GWS was used as the Investigator's Photodamage Assessment and was completed at the screening visit. Only participants with a Glogau Scale Type II (i.e., wrinkling in skin with movement) or III (wrinkles at rest) qualified for the study.

#### 2.3.4 Photographs Obtained

Photographs were taken at all visits from baseline to week 12 using the three‐dimensional Canfield Scientific Visia‐CR facial imaging system. Photographs were taken of participants with freshly cleaned and dry faces. No makeup was worn, including no foundation, blush, eye shadow, lipstick, and mascara. Participants were required to remove all jewelry for the photographs, use headbands to keep their hair away from their faces, and keep their eyes closed for all photos. Participants agreed to have their photographs taken to participate in this study. Three images were captured at the required photography time points: full face, right side at a 45‐degree angle, and left side at a 45‐degree angle. All images were transferred to Canfield Scientific for processing at the end of the study.

#### 2.3.5 Method of Treatment Assignment

A unique identification number was assigned to each participant after they had signed the ICF. Participants were enrolled in chronological order, excluding screen failures. This study was not blinded. All participants received treatment with the investigational product and ancillary supplies (i.e., cleanser and moisturizer). Participants were instructed to use the cleanser and moisturizer each morning, followed by a sunscreen SPF 30+ as needed. Participants were asked to apply two pumps of the investigational product on a clean face once each evening and to record the application in the diary to ensure compliance. A complete ingredient list for the investigational retinal can be found in Figure S3.

#### 2.3.6 Investigator Skin Quality Assessments

Complete face assessments of erythema, skin tone (discoloration), skin texture (tactile roughness), and lines/wrinkles were conducted at all visits, from baseline to week 12. These parameters were graded by the Investigator using an ordinal 6‐point scale (0 = none, 1 = minimal, 2 = mild, 3 = moderate, 4 = moderately severe, 5 = severe) at every visit, as outlined in the table of study procedures (Table 1).

**TABLE 1:** Schedule of study procedures.

	Visit 1	Visit 2	Visit 3	Visit 4	Visit 5	Visit 6
	Screening	Baseline	Week 2	Week 4	Week 8	Week 12
Eligible window (in days)	(−28 to 0)	Day 0	+/− 2	+/− 2	+/− 2	+/− 2
Informed consent form (ICF)/HIPAA/photo release	X					
Inclusion/exclusion criteria	X	X				
Medical intake form (Demographic data)	X					
Urine pregnancy test (if of childbearing potential)	X	X				
Glogau grading/fitzpatrick wrinkle grading	X					
Photography		X	X	X	X	X
Investigator objective assessment(s)		X	X	X	X	X
Investigator tolerability assessment		X	X	X	X	X
GAIS					X	X
Subjective tolerability assessment		X	X	X	X	X
Self‐assessment questionnaire		X				
Subject satisfaction questionnaire						X
Test product distribution/collection		D				C
Usage instructions		X				
diary distribution/review/collect		D	R	R	R	C
Diary distribution/review/collect	X	X	X	X	X	X
Adverse events		X	X	X	X	X

*Note:* D, distribution; *R*, review; C , collect; GAIS, global aesthetic improvement scale.

#### 2.3.7 Investigator Tolerability Assessment

The Investigator Tolerability Assessment (ITA) was conducted at all visits, from baseline to week 12. The ITA evaluated the presence of edema, dryness, and peeling according to a 5‐point ordinal scale, where 0 = none, 1 = minimal, 2 = mild, 3 = moderate, and 4 = severe.

#### 2.3.8 Subject Tolerability Assessment

The Subject Tolerability Assessment (STA) was conducted at all visits, from baseline to week 12. The STA evaluated whether participants experienced any sensations of stinging, tingling, itching, or burning, and graded the severity according to a 5‐point ordinal scale, where 0 = none, 1 = minimal, 2 = mild, 3 = moderate, and 4 = severe.

#### 2.3.9 Subjective Skin Quality Questionnaire

Participants completed the Subjective Skin Quality Questionnaire at the baseline/visit 2 (Day 0) only, before product application. The Questionnaire was submitted as a stand‐alone document.

#### 2.3.10 Product Usage Instructions and Participant Diary

Instructions included application expectations and the use of dispensed products; this was incorporated into the treatment diaries. At baseline, the study coordinator reviewed the instructions and diary with participants. The study product was applied once daily in the evening. Participants used diaries to document product application and ensure continued compliance with the protocol. Participants were given a diary at baseline, and it was reviewed and reconciled at each subsequent visit. The study coordinator collected the diaries at week 12 (EOS).

#### 2.3.11 Adverse Events

All adverse events (AEs), including serious AEs (SAEs), were documented in the participants' clinical report forms. An AE is any untoward medical occurrence experienced by a participant, regardless of whether it was considered study‐related. All AEs were assessed by the Investigator for severity (mild, moderate, severe) and relationship to the investigational product (not related, possible, probable, definite).

#### 2.3.12 Global Aesthetic Improvement Scale (GAIS)

The Investigator completed the Global Aesthetic Improvement Scale (GAIS) at weeks 8 and 12EOS after enrollment. The GAIS is a 5‐point scale used to assess full‐face aesthetic changes from baseline. This parameter represents a qualitative assessment of the overall skin condition, where 0 indicates no improvement, 1 indicates minimal improvement, 2 indicates mild improvement, 3 indicates moderate improvement, and 4 indicates marked improvement.

#### 2.3.13 Subject Satisfaction Questionnaire (EOS Only)

The Subject Satisfaction Questionnaire consisted of fourteen questions, which were answered using checkboxes. The questionnaire assessed the degree of change in various skin attributes (e.g., fine lines, pore size, glow, texture/smoothness) since the beginning of treatment. The Subject Satisfaction Questionnaire was administered only at the EOS visit.

### 2.4 Data Analyses

The Investigator assessments were based on numerical scales. Since the data were non‐parametric, the statistical comparisons were made with non‐parametric tests (e.g., Wilcoxon Signed Rank Test). Significance was determined if *p* < 0.05.

## 3 Results

### 3.1 Participant Demographics

Participants (100%) had a group mean age of 43.80 years (SD: 12.48). Participants (17/20; 85.00%) were Caucasian, Asian or Pacific Islander (2/20; 10.00%), and African American (1/20; 5.00%), with Fitzpatrick Skin Types II‐III (18/20, 90.00%) and V (2/20; 10.00%). At baseline, all subjects exhibited mild‐to‐moderate facial photodamage.

### 3.2 Investigator Objective Assessment(s)

Multiple Wilcoxon Signed‐Rank tests were used to compare participants' mean scores for erythema, skin tone, skin texture and lines/wrinkles scores at baseline and after treatment (i.e., weeks 2, 4, 8, and 12).

#### 3.2.1 Erythema

**FIGURE 1:**
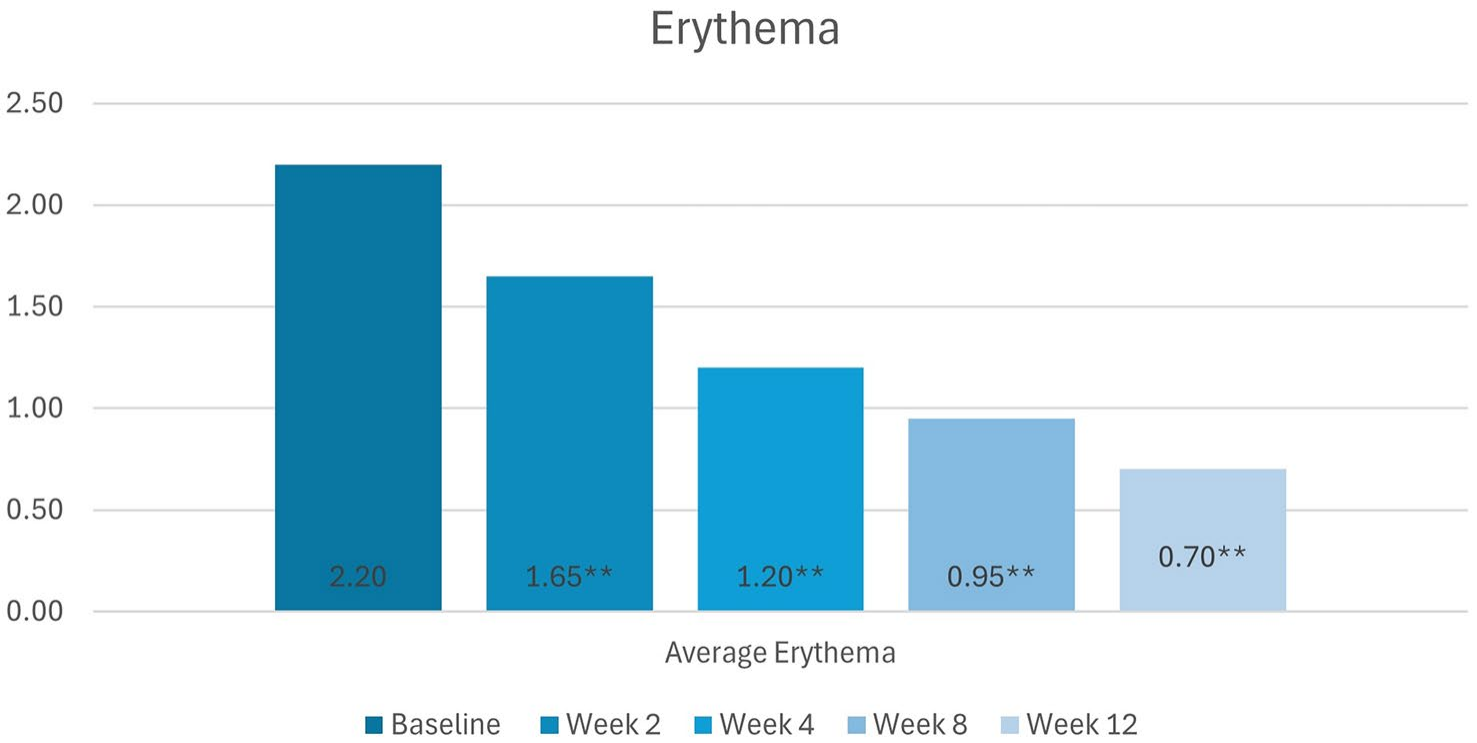
Decreases in participants' mean erythema scores (None [0] to Severe [5]) from baseline to week 12, as per the Investigator's assessments. *Source:* * = *p* < 0.05, ** = *p* < 0.01.

Statistically significant decreases in the severity of erythema from baseline were observed at all follow‐up visits (*p* < 0.01). The mean erythema scores improved from baseline at weeks 2, 4, 8, and 12 by 25.00%, 45.45%, 59.09%, and 68.18%, respectively (Figure 1).

#### 3.2.2 Skin Tone

**FIGURE 2:**
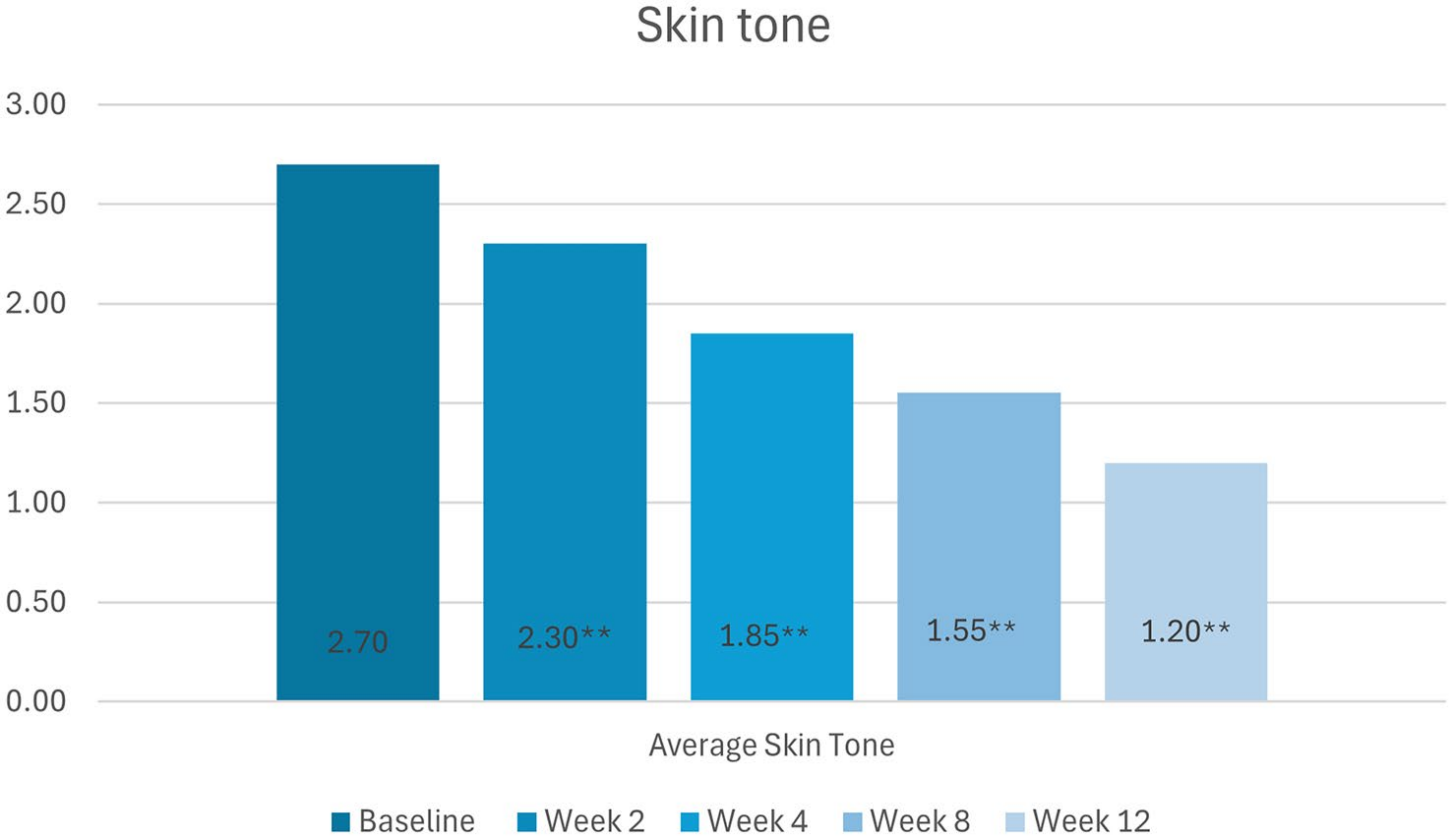
Change in participants' skin tone scores (None [0] to Severe [5]) from baseline to week 12, as per the Investigator's assessments. *Source:* * = *p* < 0.05, ** = *p* < 0.01.

Statistically significant improvements in skin tone from baseline were observed at all follow‐up visits (*p* < 0.01). Mean scores at baseline and weeks 2, 4, 8, and 12 were 2.70, 2.30, 1.85, 1.55, and 1.20, respectively (Figure 2).

#### 3.2.3 Skin Texture

**FIGURE 3:**
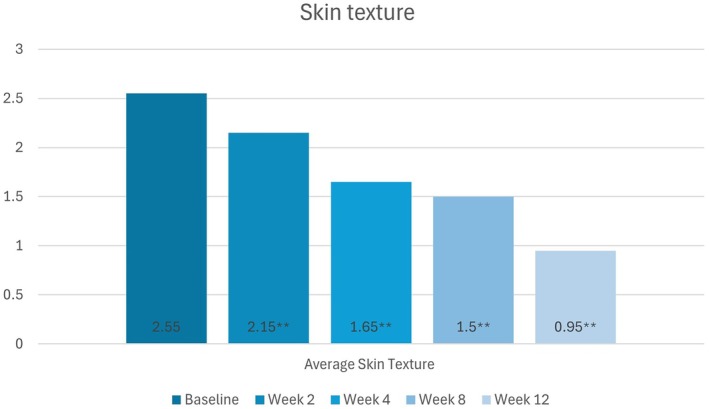
Change in participants' skin texture scores (None [0] to Severe [5]) from baseline to week 12, as per the Investigator's assessments. *Source:* * = *p* < 0.05, ** = *p* < 0.01.

Statistically significant improvements in skin texture from baseline were observed at all follow‐up visits (*p* < 0.01). Mean scores at baseline and weeks 2, 4, 8, and 12 were 2.55, 2.15, 1.65, 1.50, and 0.95, respectively (Figure 3).

#### 3.2.4 Lines/Wrinkles

**FIGURE 4:**
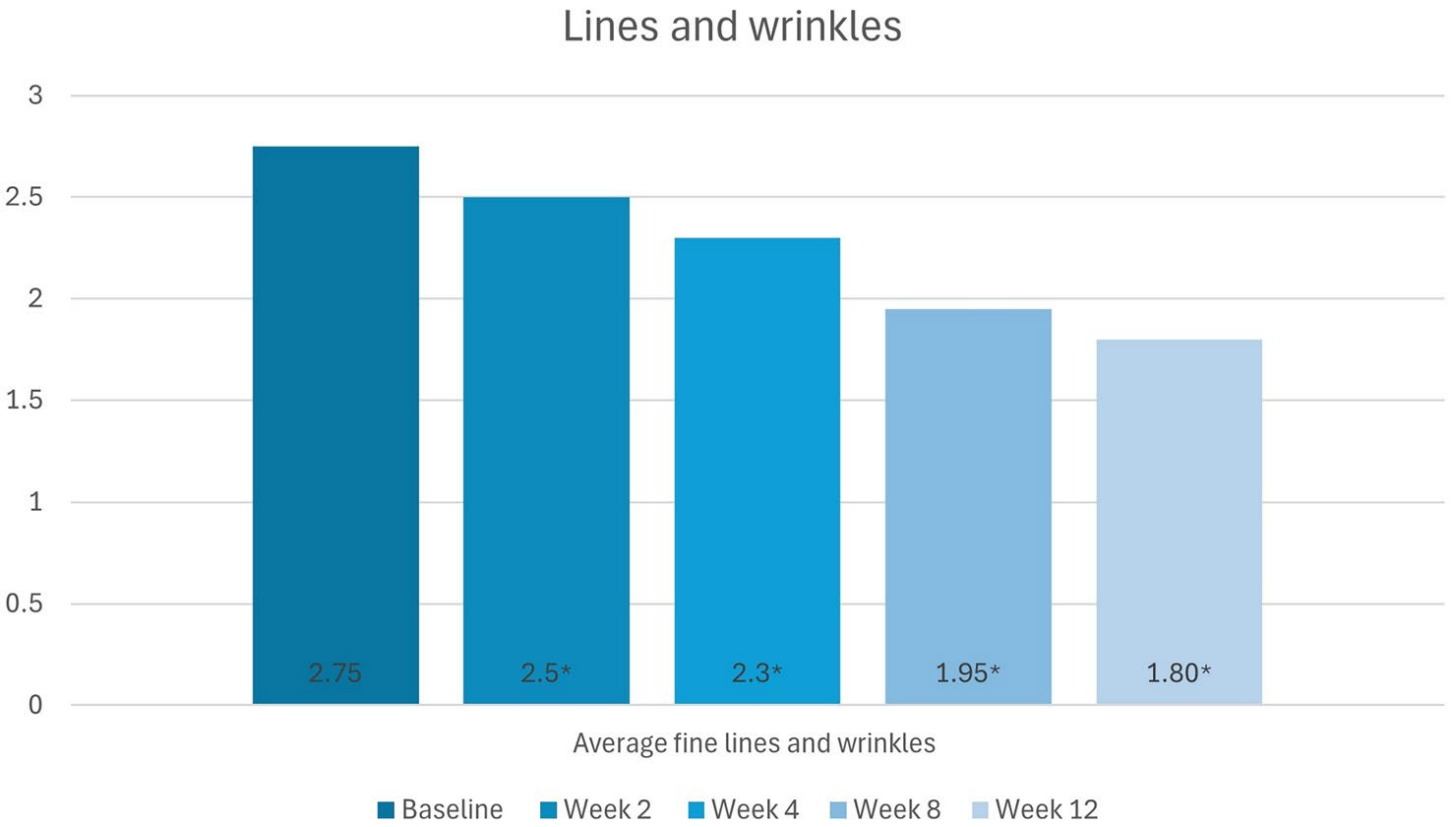
Change in participants' lines/wrinkles scores (None [0] to Severe [5]) from baseline to week 12, as per the Investigator's assessments. *Source:* * = *p* < 0.05, ** = *p* < 0.01.

Statistically significant decreases in the severity of lines/wrinkles from baseline were observed at all follow‐up visits (*p* < 0.05). Mean scores at baseline and weeks 2, 4, 8, and 12 were 2.75, 2.50, 2.30, 1.95, and 1.80, respectively (Figure 4).

### 3.3 Global Aesthetic Improvement Scale (GAIS; Weeks 8 and 12 Only)

**FIGURE 5:**
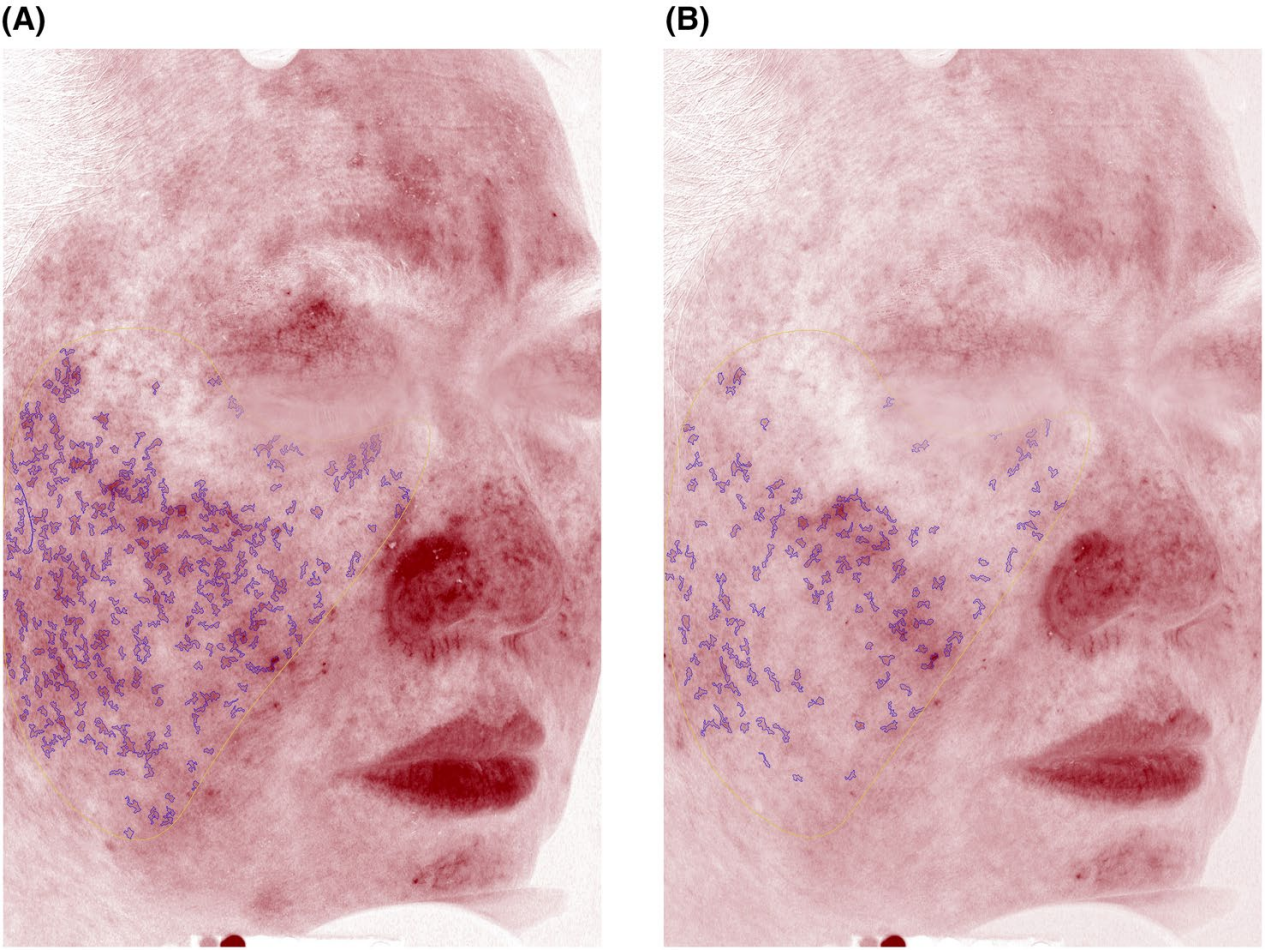
A 50‐year‐old female at baseline (left) and week 2 (right). Note that there is an overall decrease in facial erythema.

The average GAIS score at week 8 was 1.50, corresponding to a grade between “improved” and “much improved”. The average GAIS score at week 12 was 2.30, corresponding to a grade between “much improved” and very much improved (Figure 5).

### 3.4 Participant Satisfaction (Subject Satisfaction Questionnaire: Week 12 Only)

A summary of the responses (*n*) to questions #1 to 9 of the Subject Satisfaction Questionnaire is presented in Table 2. Responses to questions #10 to 14 are described in the following section. Most participants (*n* = 19/20; 95.00%) tolerated the investigational product very well, without discomfort. One participant (5.00%) reported barely noticeable, temporary discomfort. All participants (*n* = 20; 100.00%) felt the investigational product absorbed fast and was not sticky. Most participants (*n* = 15/20; 75.00%) reported that the investigational product was odorless. The remaining participants (*n* = 5; 25.00%) reported smelling an odor that they “liked much”. At the EOS, 19/20 (95.00%) participants were at least satisfied with the investigational product. Relatedly, most (19/20, 95.00%) participants reported that they would recommend the investigational product to a friend or colleague.

**TABLE 2:** Summary results of responses to questions #1 to 9 of the Subject Satisfaction Questionnaire, administered at week 12 (End of Study).

No	Question	Response frequency *N* = 20					
		Very much improved	Much improved	Slightly improved	No change	Worse	Much worse
Q1	How much have the fine lines and wrinkles improved since the start of the treatment?	2	8	8	2	0	0
Q2	How much has the visual pore size improved since the start of the treatment?	2	7	10	1	0	0
Q3	How much has the glow improved since the start of the treatment?	3	13	3	1	0	0
Q4	How much has the texture/smoothness improved since the start of the treatment?	3	13	3	1	0	0
Q5	How much has the skin tone improved since the start of the treatment?	3	9	6	2	0	0
Q6	How much has redness improved since the start of the treatment?	4	6	4	6	0	0
Q7	How much has your skin dryness improved since the start of the treatment?	4	5	8	2	1	0
Q8	How much has the youthful look improved since the start of the treatment?	3	6	10	1	0	0
Q9	How much has the overall skin quality improved since the start of the treatment?	4	11	4	0	1	0
	Note: Responses to questions 10 to 14 are described in text						

### 3.5 Tolerability Assessments and Adverse Events

Results of the ITA and STA are displayed in Tables 3 and 4, respectively. No product‐related adverse events were reported throughout the study.

**TABLE 3:** Investigator‐assessed tolerability (mean scores).

Visit	Edema	Dryness	Peeling
Baseline	0	0	0
Week 2	0	0.1	0
Week 4	0	0.05	0
Week 8	0	0.1	0
Week 12	0	0	0

**TABLE 4:** Participant‐assessed tolerability (mean scores).

Visit	Stinging	Tingling	Itiching	Burning
Baseline	0	0	0	0
Week 2	0.1	0	0.05	0.05
Week 4	0	0	0.05	0
Week 8	0.1	0	0.05	0.1
Week 12	0	0	0	0

### 3.6 Sample Cases

**FIGURE 6:**
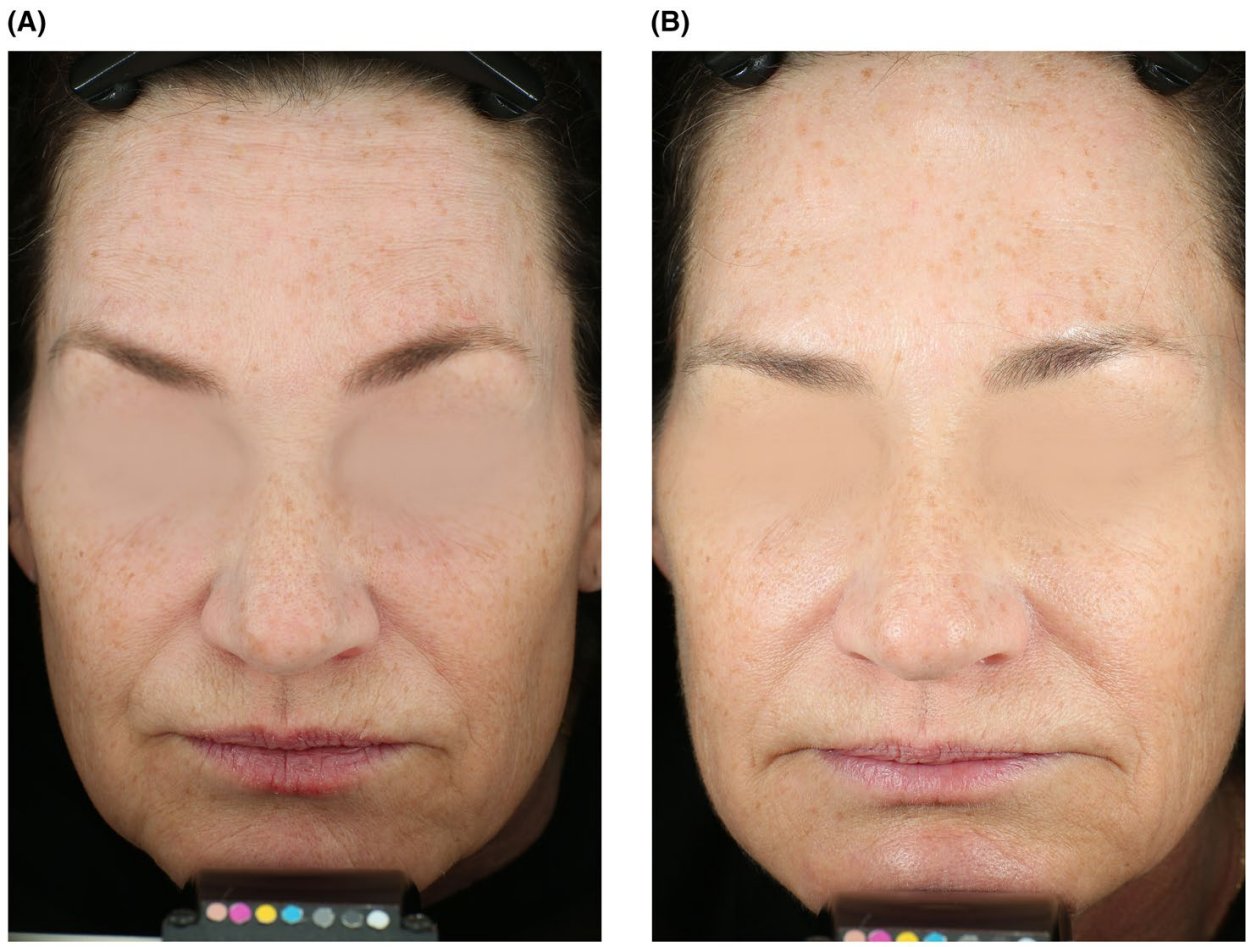
A 58‐year‐old female at baseline (left) and week 12 (right). Note that the participant exhibits improvement in wrinkles and skin quality, particularly around the perioral, periorbital, and forehead regions.

**FIGURE 7:**
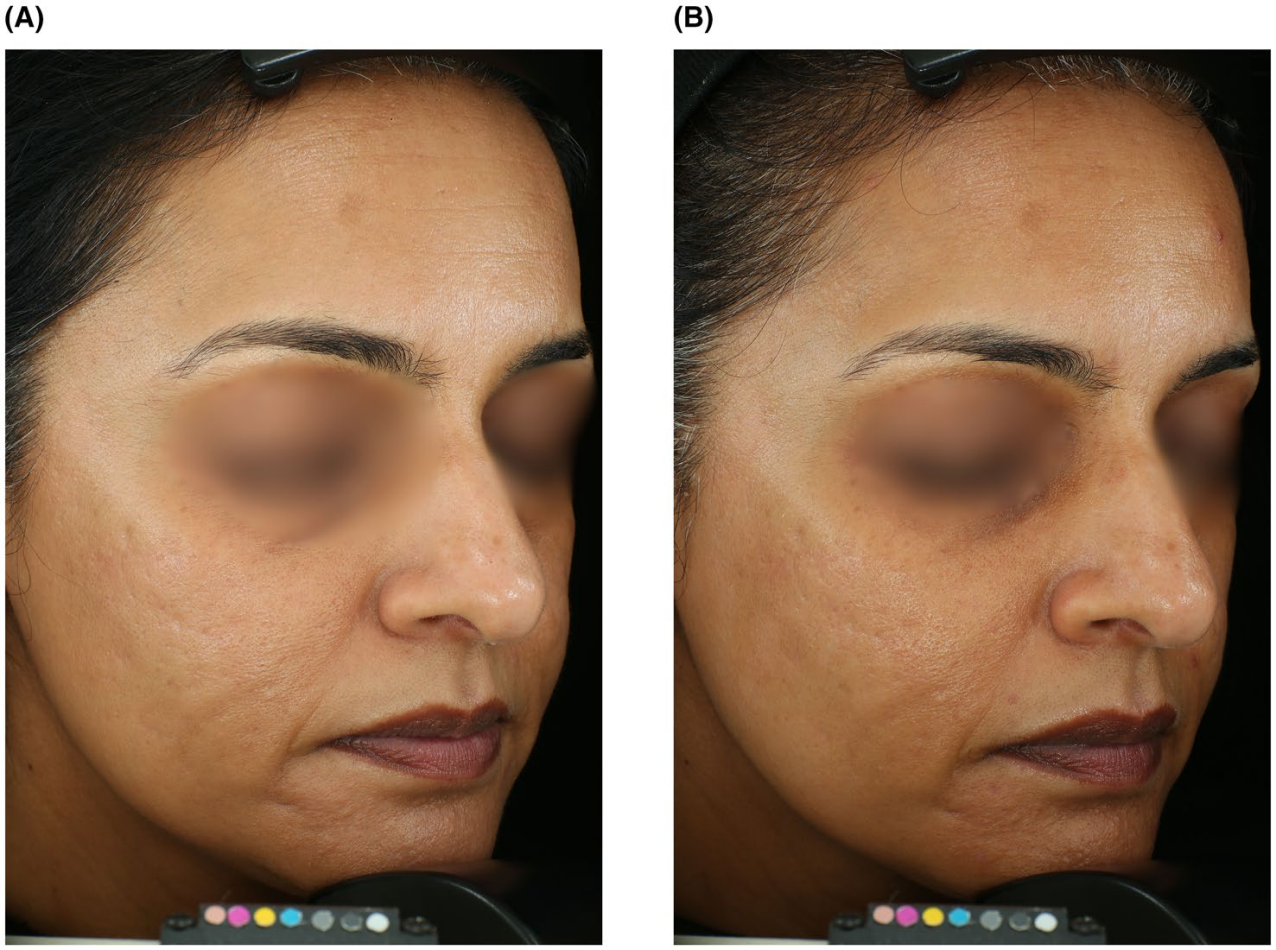
A 43‐year‐old female with skin of color at baseline and week 8. Note that the participant demonstrates improvement in acne scars, marionette lines, and forehead wrinkles.

Figure 5 displays a participant exhibiting improvement in facial erythema from baseline. Figure 6 displays a participant exhibiting improvement in wrinkles and skin quality, particularly around the perioral, periorbital, and forehead regions. Figure 7 shows a participant with skin of color who demonstrated improvement in acne scars, marionette lines, and forehead wrinkles.

## 4 Discussion

In this study, investigators evaluated the efficacy and tolerability of a novel treatment for photoaging: a retinal created using a potent HA, synergistic ingredients, and a novel delivery system. These unique ingredients and the mechanism of action allow for a high concentration of retinal to be incorporated into the formulation without causing irritation [19, 20]. Results of this study support that the proprietary formulation created a highly effective yet gentle retinal suitable for nightly use. The use of this novel retinal was associated with several aesthetic benefits, such as decreased erythema and improvements in the appearance of skin tone, skin texture, and lines/wrinkles. Moreover, it displayed a rapid onset with statistically significant improvements in parameters associated with skin health being reported by investigators and participants at week 2. Clinical improvements increased with continued use, up to week 12. The investigational product was non‐irritating and displayed a suitable safety profile throughout the 12‐week study. These results align with an earlier cohort study (data on file), which evaluated the use of the novel retinal in five participants with sensitive skin and/or rosacea. Participants were assessed after 1, 2, and 4 weeks of daily use, with evaluations focusing on tolerability and efficacy. The investigator determined that the product was well tolerated and associated with only minimal signs of irritation at weeks 1 and 2. No signs of irritation were observed by week 4.

Given the novel bioengineered delivery system and synergistic combination of ingredients, this product addresses traditional barriers to retinoid usage and may be suitable for all skin types. The formulation's innovative encapsulated delivery system and ingredient mix resulted in a rapid onset of efficacy and mitigated retinal‐associated skin irritation. Future studies should investigate the suitability of this novel retinal for sensitive skin and/or specific skin conditions (e.g., post‐acne skin, atopic dermatitis, rosacea, menopausal skin). As no participants in the present study reported periorbital or perioral dryness/dermatitis, this retinal may be appropriate for use in areas characterized by thin and/or sensitive skin. This would address a limitation of other over‐the‐counter retinoids, which are contraindicated around the eyes and mouth Future studies should explicitly evaluate this.

This study has several limitations commonly observed in clinical trials aimed at evaluating the antiaging efficacy of retinal, which may restrict the robustness of the study design, the validity of the results, and the interpretation of the conclusions. These include small sample sizes and a lack of a vehicle‐control group. Due to the small sample size used in the present trial, the study population is largely homogenous (i.e., 100% female, 90.00% Fitzpatrick Skin Type ≤ III, 85.00% Caucasian), and risks and benefits associated with specific patient subgroups may remain uncovered. For example, there are known racial and gender‐based differences in the tolerability of topical retinoids. A retrospective cohort study evaluating 753 patients prescribed topical retinoids who self‐identified as White, Black, or Asian found that the odds of intolerability were lower in the Black than in the Asian population. There was a statistically nonsignificant trend toward lower intolerance in White than in Asian patients, and for Black versus Asian populations [21]. Additionally, males exhibited better tolerance to topical retinoids than females, which is consistent with previous reports demonstrating gender‐related differences in skin physiology [22]. Moreover, although randomized, double‐blind, vehicle‐controlled studies are rare in practice [23, 24, 25, 26], they are needed as significant improvements in skin quality have been observed with vehicles alone [19] Therefore, future directions should emphasize the design of additional well‐designed studies.

## Author Contributions

Author M.H.G. led the investigation and collected the data. K.M.E. drafted the initial manuscript. All authors then critically revised the manuscript for important intellectual content and approved the final version. Authors listed alphabetically by last name indicate equal contribution.

## Funding

This work was supported by Hydrinity.

## Ethics Statement

This study was conducted in accordance with the Declaration of Helsinki and received prior approval from a research ethics board (Advarra; Pro00084505; Date: 22/Jan/2025). All participants provided written informed consent before their enrollment in the study.

## Consent

All participants displayed in this manuscript provided written consent for the use of their facial photographs.

## Conflicts of Interest

The Tennessee Clinical Research Center (Dr. Gold is the Medical Director) received research funds to perform the clinical trial.

## Supporting information


**Figure S1:** Inclusion criteria.
**Figure S2:** Exclusion criteria.
**Figure S3:** Complete ingredient list for the investigational retinol.
**Figure S4:** Graphs with Axis Labels.

## Data Availability

The data that support the findings of this study are available from the corresponding author upon reasonable request.
