# Supplementary material for: A Single‐Center, Open‐Label Study to Evaluate the Efficacy and Tolerability of Retinal Encapsulated in a Novel Biomimetic Exosome in the Treatment of Mild‐To‐Moderate Facial Photodamage
Source: J Cosmet Dermatol. 2026 Feb 24;25(3):e70755. doi: 10.1111/jocd.70755 (PMC12932260; doi:10.1111/jocd.70755)
Supplement: Supplementary file 1 — Figure S1: Inclusion criteria. Figure S2: Exclusion criteria. Figure S3: Complete ingredient list for the investigational retinol. Figure S4: Graphs with Axis Labels. [file JOCD-25-e70755-s001.docx]

**Supplementary material**

**S1.** Inclusion criteria.

**S2.** Exclusion criteria.

**S3.** Complete ingredient list for the investigational retinol.

Graphs with Axis Labels

**

**

**

**

**

**

**

**

**

**

**

**

*

*

*

*
